# The PedsQL™ as a patient-reported outcome in children and adolescents with Attention-Deficit/Hyperactivity Disorder: a population-based study

**DOI:** 10.1186/1477-7525-4-26

**Published:** 2006-04-21

**Authors:** James W Varni, Tasha M Burwinkle

**Affiliations:** 1 Department of Pediatrics, College of Medicine, Department of Landscape Architecture and Urban Planning, College of Architecture, Texas A&M University, 3137 TAMU, College Station, TX 77843-3137, USA; 2 The Children's Hospital at Scott & White, Department of Pediatrics, Texas A&M University Health Science Center, 2401 South 31^st ^Street, Temple, TX 76508, USA

## Abstract

**Background:**

Attention-Deficit/Hyperactivity Disorder (ADHD) is the most common chronic mental health condition in children and adolescents. The application of health-related quality of life (HRQOL) as a *pediatric population health *measure may facilitate risk assessment and resource allocation, the identification of health disparities, and the determination of health outcomes from interventions and policy decisions for children and adolescents with ADHD at the local community, state, and national health level.

**Methods:**

An analysis from an existing statewide database to determine the feasibility, reliability, and validity of the 23-item PedsQL™ 4.0 (Pediatric Quality of Life Inventory™) Generic Core Scales as a patient-reported outcome (PRO) measure of pediatric population health for children and adolescents with ADHD. The PedsQL™ 4.0 Generic Core Scales (Physical, Emotional, Social, School Functioning) were completed by families through a statewide mail survey to evaluate the HRQOL of new enrollees in the State of California State's Children's Health Insurance Program (SCHIP). Seventy-two children ages 5–16 self-reported their HRQOL.

**Results:**

The PedsQL™ 4.0 evidenced minimal missing responses, achieved excellent reliability for the Total Scale Score (α = 0.92 child self-report, 0.92 parent proxy-report), and distinguished between healthy children and children with ADHD. Children with ADHD self-reported severely impaired psychosocial functioning, comparable to children with newly-diagnosed cancer and children with cerebral palsy.

**Conclusion:**

The results suggest that population health monitoring may identify children with ADHD at risk for adverse HRQOL. The implications of measuring pediatric HRQOL for evaluating the population health outcomes of children with ADHD internationally are discussed.

## Background

While the importance of measuring pediatric health-related quality of life (HRQOL) in clinical trials is increasingly recognized for children with chronic health conditions [1,2], the utility of pediatric HRQOL measurement in *population health *outcome evaluation from the perspective of children in large pediatric populations has several distinct benefits *beyond the clinical setting*. It can aid in identifying subgroups of children who are at-risk for health problems, in determining the burden of a particular disease or disability, and in informing efforts aimed at prevention and intervention at the local community, state, and national level [3-5]. In addition, utilization of HRQOL measures at the *population health level *may assist in the evaluation of the healthcare needs of a community, and results can be used to influence public policy decisions, including the development of strategic healthcare plans, identifying health disparities, promoting policies and legislation related to community health, and aiding in the allocation of healthcare resources [6].

ADHD is the most common chronic mental health condition in children and adolescents [7]. Recently, a number of studies have reported on the HRQOL of children with ADHD utilizing parent proxy-reported instruments [8-14]. These investigations have made an important contribution by identifying the significant negative impact on HRQOL of ADHD in children from the perspective of caregivers. However, given that patient self-report is considered the standard in patient-reported outcomes measurement [15-18], a reliance on only parent proxy-report is insufficient. There is a critical need to empirically document the HRQOL of children with ADHD from *their *perspective, or in other words, to "hear the voices of the children" in matters pertaining to their health and well-being for the youngest children possible [19]. Several studies have investigated the HRQOL construct in ADHD from the perspective of primarily young adolescent and adolescent self-report [20,21], with the except of one study which included a combined sample of children 6–18 years of age referred for psychiatric services who were diagnosed with a variety of disorders within a spectrum of attention-deficit and disruptive behavior disorders [22].

Patient-reported outcomes (PROs) are self-report instruments that directly measure the *patient's perceptions *of the impact of disease and treatment as clinical trial endpoints, and include multi-item HRQOL instruments, as well as single-item measures (e.g., pain visual analogue scale), daily diaries, treatment adherence, and healthcare satisfaction [15,16,23]. Pediatric PROs must be sensitive to cognitive development and should include both child self-report and parent proxy-report to reflect their potentially unique perspectives. However, imperfect agreement between self and proxy report, termed cross-informant variance [24], has been consistently documented in the PRO measurement of children with chronic health conditions and healthy children [25,26]. The demonstration of cross-informant variance and the general acceptance that HRQOL derives from an individual's perceptions [17], indicates an essential need in pediatric HRQOL measurement for reliable and valid child self-report instruments for the broadest age range possible.

Although other pediatric HRQOL instruments exist, including generic measures and disease-specific measures [2,27], it has been an explicit goal of the Pediatric Quality of Life Inventory™ (PedsQL™) Measurement Model [28] to develop and test brief age-appropriate PRO measures for the broadest age group empirically feasible, specifically including child self-report for the youngest children possible [18,29]. This goal was originally articulated in empirical efforts in the 1980's to measure pain perception in pediatric patients through the development and testing of the Varni/Thompson Pediatric Pain Questionnaire™ for children as young as 5 years of age [30]. Thus, a major goal of the PedsQL™ programmatic research efforts is to document the potential for child self-report in patient populations in which proxy-report has been consider the standard for young children [19].

Consequently, the primary objective of the present study was to measure the perceived HRQOL of children with ADHD *from the perspective of the children *at the *population health level *utilizing the PedsQL™ 4.0 Generic Core Scales. The data were derived from a statewide mail survey to families with children ages 2–16 years throughout the State of California encompassing all new enrollees in the State's Children's Health Insurance Program (SCHIP) during a two month period [4].

Based on the extant literature on HRQOL in pediatric chronic health conditions in general [27], and ADHD in particular [22], we hypothesized that children with ADHD would self-report significantly lower psychosocial health than healthy children, while self-reporting only slightly lower physical health. We further examined the concordance between child self-report and parent proxy-report, expecting moderate agreement based on the extant literature with the PedsQL™ in pediatric chronic health conditions [31-33] and psychiatric disorders [34]. In order to further determine the clinical magnitude of the hypothesized negative impact of ADHD on pediatric patient self-reported HRQOL, we conducted comparative analyses between children with ADHD and children with newly-diagnosed cancer and children with cerebral palsy, both groups who have previously demonstrated significantly impaired self-reported HRQOL using the PedsQL™ [19,31].

## Method

### SCHIP sampling frame and procedure

The PedsQL™ 4.0 survey was mailed separately for each of the months of February and March 2001 to 20,031 families with children ages 2–16 years throughout the State of California, which encompassed all new enrollees in the State's Children's Health Insurance Program (SCHIP) for those months and for those ages and for parents and/or children who were English-, Spanish-, Vietnamese-, Korean-, or Cantonese-speaking [4]. The overall return rate was 51%. This response rate was expected for the mode and method of survey administration utilized, which involved a one-time only statewide mailing given limited funding from the sponsor which did not permit more follow-up contacts [35]. Although the PedsQL™ 4.0 can be administered to children ages 2–18, children older than 16 years of age were not included in this field test because a two-year follow-up was anticipated (the oldest children in the sample would be 18 years old at the 2-year follow-up). The mail survey mode of administration was paper-and-pencil self-administration for parents and children ages 8 to 16, and parent-assisted administration for children ages 5 to 7. Since we were primarily interested in child self-report for ages 5–16, we did not include analyses of data for parent proxy-report for children ages 2–4.

DataStat, a nationally-based survey administration firm located in Michigan, was contracted to administer the California SCHIP statewide mail survey. DataStat mailed the PedsQL™ 4.0 survey, together with a cover letter to all eligible SCHIP families during the two month period selected by the State of California. Parents and children were instructed in the cover letter to complete the PedsQL™ 4.0 separately, except for children ages 5–7, who were assisted by their parents after the parent completed the proxy-report. Parents were also instructed to complete the additional survey items after completing the PedsQL™ 4.0. In order to assure the anonymity and confidentiality of the respondent's answers and the neutrality of the organization gathering the data, all surveys were mailed back to DataStat. Since the intent of the SCHIP project was program evaluation and not research, parents and children did not complete informed consent forms, as described in the original report [4]. Consequently, deidentified data were analyzed for this report by the investigators. This protocol of analyzing existing deidentified data was approved by the Institutional Review Board at Children's Hospital and Health Center, San Diego.

### Healthy children SCHIP sample

The healthy children subgroup sample was derived from the PedsQL™ 4.0 SCHIP total sample [4]. The healthy sample was randomly matched by age group to the ADHD sample prior to analysis (i.e., an equal percentage of healthy children in each age group was randomly selected to match the ADHD sample by age group using the SPSS random sample case selection command). Child self-report and parent proxy-report were available for all cases. The average age of the 1693 boys (51.9 %) and 1567 girls (48.1 %) was 10.75 years (SD = 3.10). The sample was heterogeneous with respect to race/ethnicity, with 478 (14.7 %) White non-Hispanic, 1971 (60.5 %) Hispanic, 69 (2.1 %) Black non-Hispanic, 389 (11.9 %) Asian/Pacific Islander, 13 (0.4 %) American Indian or Alaskan Native, and 340 (10.4 %) missing. The statewide SCHIP sample was representative of low-income families (≤ 250% of the federal poverty level) [4].

### ADHD SCHIP sample

The ADHD subgroup sample was derived from the same PedsQL™ 4.0 SCHIP total sample as the healthy children subgroup sample [4]. For 72 children ages 5 to 16 years, child self-report was available. The average age of the 60 boys (83.3%) and 12 girls (16.7%) was 10.95 years (SD = 3.13) with a range of 5 to 16 years. The sample was heterogeneous with respect to race/ethnicity, with 31 (43.1%) White, 16 (22.2%) Hispanic/Latino, 7 (9.7%) Black/African American, 7 (9.7%) Asian/Pacific Islander, 2(2.8%) Native American or Native Alaskan, and 9 (12.5%) missing. The statewide SCHIP sample was representative of low-income families, that is, incomes at or below 250% of the federal poverty level) [4].

### Pediatric cancer sample

The cancer sample was derived from the PedsQL™ Cancer Module field test [31], and randomly matched by age group to the ADHD sample. The sample included newly-diagnosed children with acute lymphocytic leukemia (n = 44, 61.1%), brain tumor (n = 6, 8.3%) non-Hodgkin's lymphoma (n = 4, 5.6%), Hodgkin's lymphoma (n = 2, 2.8%), Wilm's tumor (n = 1, 1.4%), and other cancers (n = 15, 20.8%). For all forms combined, the average age of the 38 boys (52.8%) and 33 girls (45.8%; Missing = 1, 1.4%) was 10.35 years (SD = 3.29) with a range of 5 to 16 years. For child self-report, the average age of the 35 boys (53.0%) and 30 girls (45.5%; Missing = 1, 1.5%). was 10.65 years (SD = 3.20). The sample was heterogeneous with respect to race/ethnicity, with 24 (33.3%) White non-Hispanic, 35 (48.6%) Hispanic, 3 (4.2%) Black non-Hispanic, 1 (1.4%) Asian/Pacific Islander, 1 (1.4%) American Indian or Alaskan Native, and 8 (11.1%) missing. Mean socioeconomic status (SES) was 32.45 (SD = 15.61), indicating on average a lower middle class sample based on the Hollingshead index[36].

### Pediatric cerebral palsy sample

The cerebral palsy sample was derived from the PedsQL™ Cerebral Palsy Module field test [37], and randomly matched by age group to the ADHD sample. The sample included children with hemiplegia (N = 22 (37.9%), diplegia (N = 22, 37.9%), and quadriplegia (N = 8, 13.8%; Missing = 6, 10.3%). Child self-report and parent proxy-report were available for all cases. For all forms combined, the average age of the 30 boys (51.7%) and 27 girls (46.6%; Missing = 1, 1.7%) was 9.79 years (SD = 3.14) with a range of 5 to 16 years. The sample was heterogeneous with respect to race/ethnicity, with 23 (39.7%) White non-Hispanic, 17 (29.3%) Hispanic, 5 (8.6%) Black non-Hispanic, 5 (8.6%) Asian/Pacific Islander, and 8 (13.8%) missing. Mean socioeconomic status (SES) was 40.33 (SD = 14.45), indicating on average a lower middle to middle class sample based on the Hollingshead index [36].

## Measures

### The PedsQL™ 4.0 (Pediatric quality of Life inventory™ Version 4.0)

The 23-item PedsQL™ 4.0 Generic Core Scales encompass: 1) Physical Functioning (8 items), 2) Emotional Functioning (5 items), 3) Social Functioning (5 items), and 4) School Functioning (5 items), and were developed through focus groups, cognitive interviews, pre-testing, and field testing measurement development protocols [29]. The instrument takes approximately 5 minutes to complete [29]. The child self-report items are contained in the Appendix.

The PedsQL™ 4.0 Generic Core Scales are comprised of parallel child self-report and parent proxy-report formats. Child self-report includes ages 5–7, 8–12, and 13–18 years. Parent proxy-report includes ages 2–4 (toddler), 5–7 (young child), 8–12 (child), and 13–18 (adolescent), and assesses parent's perceptions of their child's HRQOL. The items for each of the forms are essentially identical, differing in developmentally appropriate language, or first or third person tense. The instructions ask how much of a problem each item has been during the past one month. A 5-point response scale is utilized across child self-report for ages 8–18 and parent proxy-report (0 = never a problem; 1 = almost never a problem; 2 = sometimes a problem; 3 = often a problem; 4 = almost always a problem). To further increase the ease of use for the young child self-report (ages 5–7), the response scale is reworded and simplified to a 3-point scale (0 = not at all a problem; 2 = sometimes a problem; 4 = a lot of a problem), with each response choice anchored to a happy to sad faces scale.

Items are reverse-scored and linearly transformed to a 0–100 scale (0 = 100, 1 = 75, 2 = 50, 3 = 25, 4 = 0), so that higher scores indicate better HRQOL. Scale Scores are computed as the sum of the items divided by the number of items answered (this accounts for missing data). If more than 50% of the items in the scale are missing, the Scale Score is not computed. This accounts for the differences in sample sizes for scales reported in the Tables. Although there are other strategies for imputing missing values, this computation is consistent with the previous PedsQL™ peer-reviewed publications, as well as other well-established HRQOL measures [29,38,39]. For this study, over 95% of child and parent respondents were included in the Scale Score analyses after imputing missing values. The Physical Health Summary Score (8 items) is the same as the Physical Functioning Subscale. To create the Psychosocial Health Summary Score (15 items), the mean is computed as the sum of the items divided by the number of items answered in the Emotional, Social, and School Functioning Subscales.

### Additional survey items

The parents completed several additional survey items adapted from the PedsQL™ Family Information Form [29]. One survey question asked the parent to report on the presence of a chronic health condition ("In the past 6 months, has your child had a chronic health condition?") defined as a physical or mental health condition that has lasted or is expected to last at least 6 months and interferes with the child's activities. If the parents check "Yes" to this question, they were asked "Which of the following chronic illnesses does your child suffer from?" The list provided consisted of asthma, diabetes, attention deficit/hyperactivity disorder (ADHD), depression, and other (write in). Parents who selected ADHD comprised the sample reported herein.

### Statistical analysis

The feasibility of the PedsQL™ 4.0 as a population health measure in ADHD was determined from the percentage of missing values for each item [29,38,39]. Scale internal consistency reliability was determined by calculating Cronbach's coefficient alpha. Scales with reliabilities of 0.70 or greater are recommended for comparing patient groups, while a reliability criterion of 0.90 is recommended for analyzing individual patient scale scores [40].

Construct validity was determined utilizing the known-groups method. The known-groups method compares scale scores across groups known to differ in the health construct being investigated. In this study, analysis of variance with Tukey post-hoc tests was used to compare groups differing in known health status (ADHD versus cancer, CP, and healthy). Although the Tukey post-hoc test is among the more conservative post-hoc approaches (therefore reducing the likelihood of Type I error), a Bonferroni correction was applied to account for multiple comparisons (in this study, 4 groups × 6 summary scores = 24/.05 = 0.002 adjusted alpha level for significance). In order to determine the magnitude of the differences between children with ADHD and healthy children, effect sizes were calculated [41]. Effect size as utilized in these analyses was calculated by taking the difference between the healthy sample mean and the ADHD sample mean, divided by the healthy sample standard deviation. Effect sizes for differences in means are designated as small (.20), medium (.50), and large (.80) in magnitude [41]. We also explored similarities and differences in PedsQL™ scores between the ADHD sample with the cancer and cerebral palsy samples.

The concordance between patient self-report and parent proxy-report was determined through correlation coefficients. Pearson Product Moment Correlation coefficient effect sizes are designated as small (.10–.29), medium (.30–.49), and large (≥50) [41]. Intraclass correlations (ICC) were also computed, designated as ≤0.40 poor to fair agreement, 0.41–0.60 moderate agreement, 0.61–0.80 good agreement, and 0.81–1.00 excellent agreement [42,43].

Statistical analyses were conducted with SPSS. Response equivalence has been previously demonstrated across language for the PedsQL™ by examining the percent missing data, floor and ceiling effects, and scale internal consistency across language, as well as across mode of administration [29]. Responses were therefore pooled (i.e., analyses included responses on all language forms of the PedsQL™).

## Results

### Feasibility

To assess instrument feasibility, the percentage of missing values was calculated. For child self-report and parent proxy-report, the percentage of missing item responses for the ADHD sample was 0.0% and 4.9%, respectively. The majority of the missing item responses for parent proxy-report were a function of 3 parents who did not complete enough items to derive scale or summary scores.

### Descriptive statistics

Table 1 presents the means and standard deviations of the PedsQL™ 4.0 scores for child self-report and parent proxy-report. The mean ADHD scale scores for the survey sample are generally consistent with the PedsQL™ 4.0 scores for a clinic sample of newly-referred pediatric patients with a physician-derived mixed diagnostic group which included ADHD in The Netherlands [22].

**Table 1:** PedsQL™ 4.0 Generic Core Scales Scores for Child Self-Report and Parent Proxy-Report across Samples

	ADHD^a^		Cancer^b^		Cerebral Palsy^c^		Healthy^d^		Differences	Effect Size
Scale	Mean	SD	Mean	SD	Mean	SD	Mean	SD		

**Self-Report**	**(N = 72)**		**(N = 66)**		**(N = 57)**		**(N = 3256)**			
Total Score	70.17	18.28	68.95	15.15	66.27	15.90	84.29	12.56	a<d***	1.12
Physical Functioning	82.63	17.47	65.81	20.33	64.81	21.36	88.02	13.26	b, c<a***; a<d**	.41
Psychosocial Health	63.52	21.05	70.81	15.31	67.00	16.08	82.31	13.95	a<b*; a<d***	1.35
Emotional Functioning	65.27	25.74	68.94	20.52	66.16	23.34	79.45	18.00	a<d***	0.79
Social Functioning	65.55	28.12	78.64	18.38	70.18	18.94	85.95	16.49	a<b***; a<d***	1.24
School Functioning	59.76	21.23	64.26	19.64	64.62	21.11	81.50	16.10	a<d***	1.35
**Proxy-Report**	**(N = 69)**		**(N = 71)**		**(N = 57)**		**(N = 3251)**			
Total Score	69.50	16.17	60.67	19.09	56.27	17.27	79.87	16.24	b<a**; c<a***; a<d***	0.64
Physical Functioning	84.61	15.89	56.75	26.34	53.31	24.28	81.76	20.82	b, c<a***	0.14
Psychosocial Health	61.43	18.79	63.12	17.68	57.85	16.71	78.85	16.00	a<d***	1.09
Emotional Functioning	64.36	21.16	58.10	21.96	61.40	18.70	79.51	17.32	a<d***	0.87
Social Functioning	65.11	26.92	71.23	19.62	54.74	21.64	80.98	20.85	c<a*; a<d***	0.76
School Functioning	53.83	19.10	61.42	21.59	57.39	19.86	76.01	19.66	a<d***	1.13

Note: ADHD = Attention-Deficit/Hyperactivity Disorder. SCHIP = State's Children's Health Insurance Program. Three parents of children with ADHD did not complete enough items to calculate subscale or summary scores.

*p < .05, **p < .01, ***p < .001 based on Tukey post-hoc analysis. Effect sizes are designated as small (.20), medium (.50), and large (.80). Effect sizes represent the magnitude in the differences between the ADHD and healthy samples only.

With a Bonferroni correction for the number of comparisons, differences at p < .05 and .01 should be considered heuristic/exploratory.

### Internal consistency reliability

Internal consistency reliability alpha coefficients by age group are presented in Table 2. The majority of the child self-report scales and parent proxy-report scales exceeded the minimum reliability standard of 0.70 required for group comparisons, while the Total Scale Score across the ages approached or exceeded the reliability criterion of 0.90 recommended for analyzing individual patient scale scores.

**Table 2:** PedsQL™ 4.0 Generic Core Scales Internal Consistency Reliability for Child Self-Report and Parent Proxy-Report for ADHD Sample by Age and Summary Score/Subscale

Scale	Age Group			
	
	Young Child 5–7	Child 8–12	Adolescent 13–18	**Total Sample**
**Child Self-Report**	**(n = 13)**	**(n = 37)**	**(n = 22)**	**(n = 72)**
Total Score	0.86	0.93	0.94	**0.92**
Physical Functioning	0.63	0.84	0.89	**0.83**
Psychosocial Health	0.86	0.91	0.92	**0.90**
Emotional Functioning	0.83	0.89	0.85	**0.87**
Social Functioning	0.85	0.89	0.90	**0.88**
School Functioning	0.52	0.70	0.82	**0.74**
				
**Parent Proxy-Report**	**(n = 13)**	**(n = 35)**	**(n = 21)**	**(n = 69)**
Total Score	0.80	0.92	0.94	**0.92**
Physical Functioning	0.61	0.87	0.78	**0.81**
Psychosocial Health	0.83	0.89	0.95	**0.90**
Emotional Functioning	0.79	0.85	0.85	**0.84**
Social Functioning	0.78	0.90	0.91	**0.88**
School Functioning	0.54	0.70	0.88	**0.76**

### Construct validity

Table 1 contains the PedsQL™ 4.0 scores for children with ADHD and healthy children within the SCHIP sample. Consistent with previous research [22], children with ADHD scored significantly lower on the Psychosocial Health scales than healthy children, with smaller differences observed on the Physical Functioning Scale. Most effect sizes were in the large range.

### Comparison to pediatric cancer and cerebral palsy

Table 1 demonstrates that children with ADHD in this sample reported psychosocial health comparable to child and parent reports of children with newly-diagnosed children receiving cancer treatment and children with cerebral palsy.

### Parent/child concordance

Table 3 shows the intercorrelations between PedsQL™ 4.0 child self-report and parent proxy-report. The overall scale intercorrelations are generally consistent with other PedsQL™ 4.0 studies [31-33], with most effect sizes in the medium to large range.

**Table 3:** Intercorrelations between PedsQL™ 4.0 Generic Core Scales Child Self-Report and Parent Proxy-Report for ADHD Sample

PedsQL™ Scale Score	Intercorrelations
Total Scale Score	0.71*** 0.70***
Physical Functioning	0.67*** 0.67***
Psychosocial Health	0.69*** 0.69***
Emotional Functioning	0.67*** 0.66***
Social Functioning	0.75*** 0.75***
School Functioning	0.59*** 0.59***

***p < .001.

Effect sizes are designated as small (.10), medium (.30), and large (.50) for Pearson Product Moment correlations.

Intraclass correlations (ICC) are designated as ≤0.40 poor to fair agreement, 0.41–0.60 moderate agreement, 0.61–0.80 good agreement, and 0.81–1.00 excellent agreement. Single Measure Intraclass Correlation Coefficients (ICC) are listed in italics below Pearson Product Moment correlation values. ICC values were derived using a single measure model.

## Discussion

These analyses from an existing database support the feasibility, reliability and validity of the PedsQL™ 4.0 as a child self-report and parent proxy-report HRQOL measurement instrument for *pediatric population health monitoring *for children and adolescents with ADHD. Items on the PedsQL™ 4.0 had minimal missing responses, suggesting that children and parents are willing and able to provide good quality data regarding the child's HRQOL at the population health level.

The PedsQL™ 4.0 self-report and proxy-report internal consistency reliabilities generally exceeded the recommended minimum alpha coefficient standard of 0.70 for group comparisons. The PedsQL™ 4.0 Generic Core Scales Total Score and the Psychosocial Health Summary Score for child self-report and parent proxy-report approached or exceeded an alpha of 0.90, recommended for individual patient analysis [40], making the Total Scale Score suitable as a summary score for the primary analysis of HRQOL outcome in population health analyses for children with ADHD, with the PedsQL™ Psychosocial Health Summary Score suitable alternatively as either the primary or secondary outcome score depending on the intent of a particular clinical trial.

As hypothesized, children with ADHD self-reported significantly lower PedsQL™ scores on dimensions of psychosocial health and slightly lower but not statistically significant differences in physical functioning in comparison to healthy children. These findings are consistent with PedsQL™ ADHD findings from The Netherlands [22] and Thailand [44]. These multinational consistencies support the potential international generalizability of these findings. It should be noted that these findings are not consistent with a study which found no differences between healthy children and children with ADHD using the CHQ self-report version [21], which may reflect age and instrument differences or true inconsistencies with the current findings using the PedsQL™. However, given the number of proxy-reported differences between healthy children and children with ADHD reported in the literature, we believe the consistency of the present findings with the PedsQL™ of differences between healthy children and children with ADHD with *both *child self-report and parent proxy-report support a true difference. Once again, this illustrates the benefits of the PedsQL™ Measurement Model in which both child self-report and parent proxy-report are advocated [28].

The comparisons between children with ADHD with children with newly-diagnosed cancer and those children with cerebral palsy are useful in understanding the relative impact of ADHD on HRQOL. The extant literature on the adaptation of children with chronic physical health conditions demonstrates that children with chronic physical health conditions are reported to not only experience lower physical functioning, but also manifest lower emotional, social, and school functioning in comparison to healthy children [45]. Thus, the findings that children with ADHD, a chronic mental health condition, report psychosocial health comparable to children receiving chemotherapy and radiation for the treatment of newly-diagnosed cancer and children with cerebral palsy who are able to self-report their psychosocial functioning, provide further insight into the comparative impact of these pediatric chronic health conditions on HRQOL. The additional strength of these findings are that they make conceptual sense as well, given that the children with ADHD in the present study, while reporting comparable psychosocial health to children with cancer and cerebral palsy, reported significantly better physical functioning in comparison to these children with severe chronic physical health conditions.

These findings with the PedsQL™ 4.0 have potential implications for the healthcare needs of children with ADHD. Given that these children were newly enrolled in a state health insurance program for poor families, it seems reasonable to assume that they did not have prior regular access to healthcare at the time of their enrollment. In fact, the similarity of these findings to children newly-referred to a hospital-based psychiatry clinic in The Netherlands suggests that children with ADHD who are not yet receiving regular treatment may be at significant risk for considerable psychosocial health impairment, and to a lesser extent, physical health impairment. The immediate and long-term consequences of untreated or under-treated ADHD can be quite severe for children, their families, and society, given previous research which has demonstrated that ADHD severity is associated with great comorbid psychopathology [7].

The challenge for healthcare systems, States and Nations is to identify and enroll children with ADHD in evidence-based quality comprehensive healthcare services in order to mitigate these potential long-term negative consequences on child HRQOL. Given that stimulant medications have emerged as the first line of effective therapy for the treatment of ADHD [46], trials which evaluate the impact of stimulant medications on HRQOL outcomes are indicated [47].

Finally, while self-report is considered the standard for measuring perceived HRQOL, it is typically parents' perceptions of their children's HRQOL that influences healthcare utilization [48-50]. Thus, the imperfect agreement observed between child self-report and parent proxy-report supports the need to measure the perspectives of both the child and parent in evaluating pediatric HRQOL since these perspectives may be independently related to healthcare utilization and risk factors. The availability of a validated parent proxy-report measure in pediatric population health provides the opportunity to estimate child HRQOL when the child is either unable or unwilling to complete the HRQOL measure, or as proxy information when young child self-report scale reliabilities do not achieve the 0.70 standard. Although the intercorrelations between child and parent report across the physical, emotional, social, and school domains might be expected to follow the conceptualization that more observable domains (i.e., physical functioning) would yield higher intercorrelations, this has not necessary been the case in either PedsQL™ publications across various pediatric chronic health conditions, nor the published literature with other HRQOL instruments. In a comprehensive review, Eiser [27] found mixed results in terms of higher intercorrelations between self and proxy report of physical functioning across pediatric HRQOL instruments, with most studies demonstrating this effect, while some others did not. For previous PedsQL™ 4.0 publications, we have generally found higher self and proxy report intercorrelations for physical functioning in comparison to the other domains, although these differences have not been large, except for children with arthritis and other rheumatologic conditions and children with cerebral palsy in which physical functioning is a salient concern [33,37]. For children with diabetes, in which physical functioning is not as salient a concern, the intercorrelation between self and proxy report on the physical functioning scale was not the highest intercorrelation [51]. Thus, the findings across PedsQL™ studies appear consistent with the extant pediatric HRQOL literature across different instruments in regards to the effect sizes of the intercorrelations between the physical functioning and other relevant HRQOL domains, while the present findings with a chronic mental health condition suggest rather similar patient/parent concordance across the physical and psychosocial dimensions.

The present findings have several potential limitations. Given that this was a population-based mail survey, there are no guarantees that the children and parents independently completed the PedsQL™. However, if that bias existed, it would be anticipated that the bias would be equally distributed across the healthy children and children with ADHD. Parents reported on their children's chronic health conditions for the SCHIP evaluation in general, including the presence of an ADHD diagnosis for the purposes of the present analysis. Objective measures of chronic health condition would strengthen the validation process. However, in previous PedsQL™ 4.0 clinical research in pediatric patients with cancer, cardiac and rheumatic chronic health conditions, and more specifically, children with psychiatric disorders, objective medical diagnosis of these chronic diseases demonstrated similar differences between healthy children and children with ADHD, psychiatric disorders, and with chronic health conditions as shown in the present findings. Nevertheless, we are now conducting PedsQL™ research with physician-diagnosed ADHD to further extend these findings to the clinic setting. Finally, while the ADHD and healthy samples were derived from the same population sample, the cancer and cerebral palsy samples are from a clinic-based sample which may represent differences in terms of SES. However, the differences between the generally lower middle class clinic samples and the population sample are not large, but future research will need to match or control for these potential differences, including gender differences.

## Conclusion

These PedsQL™ 4.0 findings demonstrate the feasibility and measurement properties required for community and general population health survey research and evaluation for children with ADHD. Measuring perceived health from the perspective of children and their parents provides a level of accountability consistent with the Institute of Medicine report on the quality of care [52]. Additionally, population-wide monitoring has been recommended for addressing socioeconomic, racial, and ethnic disparities in healthcare quality [53]. As the consumers of pediatric healthcare, children with ADHD and parents are uniquely positioned to give their perspectives on healthcare quality through their perceptions of child health-related quality of life outcomes.

## Abbreviations

HRQOL Health-Related Quality of Life

PedsQL™ Pediatric Quality of Life Inventory™

PRO Patient-Reported Outcomes

## Competing interests

The author(s) declare that they have no competing interests.

## Authors' contributions

JWV conceptualized the rationale and design of the study. JWV designed the instrument and drafted the manuscript. TMB participated in study conceptualization and design, and performed the statistical analysis. All authors read and approved the final manuscript.

**Table 4:** Appendix A PedsQL™ 4.0 Generic Core Scales Child Self-Report Item Content

**Physical Functioning Scale**
1. It is hard for me to walk more than one block
2. It is hard for me to run
3. It is hard for me to do sports activity or exercise
4. It is hard for me to lift something heavy
5. It is hard for me to take a bath or shower by myself
6. It is hard for me to do chores around the house
7. I hurt or ache
8. I have low energy
**Emotional Functioning Scale**
1. I feel afraid or scared
2. I feel sad or blue
3. I feel angry
4. I have trouble sleeping
5. I worry about what will happen to me
**Social Functioning Scale**
1. I have trouble getting along with other kids
2. Other kids do not want to be my friend
3. Other kids tease me
4. I cannot do things that other kids my age can do
5. It is hard to keep up when I play with other kids
**School Functioning Scale**
1. It is hard to pay attention in class
2. I forget things
3. I have trouble keeping up with my schoolwork
4. I miss school because of not feeling well
5. I miss school to go to the doctor or hospital

Reproduced with permission from J.W. Varni, Ph.D. Copyright ^© ^1998.

PedsQL™ is available at
